# Temporal trends in the surgical outcomes of patients with breast cancer

**DOI:** 10.1186/1477-7819-10-108

**Published:** 2012-06-14

**Authors:** Takeshi Hanagiri, Yoshika Nagata, Shoko Monji, Shinji Shinohara, Masaru Takenaka, Yoshiki Shigematsu, Hidehiko Shimokawa, Makoto Nakagawa, Hidetaka Uramoto, Tomoko So, Fumihiro Tanaka

**Affiliations:** 1 Second Department of Surgery, School of Medicine, University of Occupational and Environmental Health, Kitakyushu, 807, Japan; 2 Second Department of Surgery, School of Medicine, University of Occupational and Environmental Health, Yahatanishi, Kitakyushu, 807, Japan

**Keywords:** Temporal trend, Surgical resection, Breast cancer, Surgical outcome

## Abstract

**Background:**

The incidence of breast cancer has been increasing in Japan over the past three decades, and it is the currently the most common malignancy in Japan. This study investigated the temporal trends of the surgical outcomes in patients with breast cancer.

**Methods:**

We evaluated 543 consecutive patients who underwent breast-cancer resection between 1980 and 2009. The temporal trends in the surgical outcome and clinicopathological features were evaluated separately for the periods covering 1980 to 1989, 1990 to 1999, and 2000 to 2009.

**Results:**

The number of patients who underwent resection during these three respective periods were 133, 176, and 234, respectively. All patients were women. The percentage of patients at stages 0 or 1 was 63.2%, 58.5%, and 43.6%, respectively, during the three periods. The mean diameter of tumors in each period was 38, 29, and 30 mm, respectively. The percentage of tumors with positive ER expression was 62.5%, 64.3%, and 69.7%, respectively. In terms of surgical procedures, the use of Halsted’s radical mastectomy decreased during each period: from 40.6% of cases to 8.5% and then to 0.4%, while the proportion of breast-conserving therapies increased, from 0% to 12.5%, and finally to 35.9%. The postoperative 10-year survival rates during the three periods were 75.9%, 83.5%, and 84.9%, respectively. The 10-year survival rates of patients with stage II disease during the three periods were 66.2%, 75.7%, and 90.7%, respectively. The prognosis of stage III disease in the three periods also showed a tendency toward improvement, increasing from 37.8% to 64.2%, and finally to 84.5%.

**Conclusion:**

The survival of patients with stage II and III disease has improved during the past 30 years. Along with the recent advances in drug therapy, the surgical treatment has become less invasive, often because of drug therapy-related modifications.

## Background

The incidence of breast cancer has been increasing in Japan over the past three decades, and it is the most common malignancy affecting women in the USA and Europe [1,2]. According to the Monitoring of Cancer Incidence in Japan project, the age-standardized incidence rate in Japan (per 100 000 population) was 17.0 in 1975, but it increased steeply to 44.4 in 2005[3].

Breast cancer is a heterogeneous disease and it has various histological types, which reflect not only morphological features, but also diverse biological characteristics. Its clinical progression is not easy to predict using the current prognostic factors. Despite advances in early detection and better understanding of the molecular basis of breast-cancer biology, about 30% of patients with breast cancer who undergo surgical resection eventually develop recurrent disease [4].

The treatment of breast cancer has changed from that advocated by Halsted, who suggested that ‘breast cancer is a local disorder involving the general body through the axillary gland and a primary area and could be cured by more expansive surgery’, to that proposed by Fisher *et al*., who considered that breast cancer is ‘a systemic disorder of which the prognosis depends on the control of micrometastases distributed throughout the general body in the early stage’ [5,6]. With the new paradigm, less invasive surgical procedures have been established. It is now considered important to administer multidisciplinary therapies, including surgery, drug treatment, and radiotherapy, to match the individual patient’s condition. The treatment strategy is based on the biological behavior of the breast cancer. Molecular targeting therapies have recently become available, and tailored treatments based on individual biological factors have already begun to play an important role in breast-cancer treatment. The correct role of surgery in multidisciplinary treatment needs to be understood, especially given the drastic changes in the trends in the treatment of breast cancer.

In this study, we retrospectively investigated the clinicopathological features of patients with breast cancer who underwent surgery, and reviewed the temporal trends in the surgical outcomes.

## Methods

We retrieved the hospital records of 543 consecutive patients who underwent resection for breast cancer between 1980 and 2009 at the Second Department of Surgery at the University of Occupational and Environmental Health. The clinical data, pre-operative examination results, details of the surgical procedure, histopathologic findings, and TNM (tumor, node, metastasis) stage of all patients were reviewed.

As part of the pre-operative evaluation, all patients had undergone physical examination, breast ultrasonography and mammography. According to the medical records, the assessments for distant metastasis included chest radiography, bone scintigraphy, and computed tomography (CT) of the chest and upper abdomen. All resected specimens, including the primary tumor and resected regional lymph nodes, were examined for tumor histology and the extent of lymph-node metastases. The histopathological findings were classified in accordance with the General Rules for Clinical and Pathological Recording of Breast Cancer 2005 proposed by the Japanese Breast Cancer Society [7]. The estrogen receptor (ER) and progesterone receptor expression levels in the cancer tissues were measured by enzyme immunoassay or by immunohistochemical staining of sections taken from formalin-fixed, paraffin wax-embedded blocks of the surgical specimens. Cancer tissue samples from 312 patients (75.5%) were available for evaluation of the hormone receptor status.

Follow-up information was obtained from all patients through office visits or telephone interviews with the patient, a relative of the patient, or the patient’s primary physician. The patients received a physical examination every 3 months for the first 2 years. Ultrasonography of the breast, chest CT, and bone scintigraphy were performed every 6 months for the first 2 years after surgery, and annually thereafter.

This research is retrospective study in very long periods. This research is in compliance with the Helsinki Declaration. However, it is impossible to obtain a signed consent form from all patients of 30 years ago.

### Statistical analysis

The survival curves were calculated by the Kaplan-Meier method and compared by using the log-rank test for univariate analysis. Categorical variables were compared by Fisher’s exact test., and *P* < 0.05 was considered significant. The Statview V software program (Abacus Concept, Berkeley, CA, USA) was used for all statistical analyses.

## Results

The temporal trends of the surgical outcomes and clinicopathological features were evaluated separately for the periods 1980 to 1989, 1990 to 1999, and 2000 to 2009. The mean age of the patients in each period was 52.1, 52.1, and 59.1 years old, respectively, and all patients were women. The number of patients who underwent a resection during these three respective periods was 133, 176, and 234, respectively (Table 1). The changes in the histological diagnoses are also shown in Table 1. The percentage of the patients without lymph-node metastasis (N0) in each period was 62.4%, 56.3%, and 64.5%, respectively, and the percentage of patients at stages 0 or 1 in each period was 63.2%, 58.5%, and 43.6%, respectively. The diameter of the tumors in each period was 38, 29, and 30 mm, respectively. The percentage of tumors with positive ER expression was 62.5%, 64.3%, and 69.7%, respectively. In terms of the surgical procedures, the use of Halsted’s radical mastectomy remarkably decreased over time, from 40.6% of procedures in the first period, to 8.5% and finally 0.4% (Table 2), while the proportion of breast-conserving therapies increased from 0% to 12.5% and finally to 35.9%. More recently, breast-conserving therapy has been performed for more than 50% of the patients with breast cancer who have undergone surgery in our hospital.

**Table 1:** The characteristics of the patients who underwent surgical resection for breast cancer

	**1980 to 1989, n = 133**	**1990 to 1999, n = 176**	**2000 to 2009, n = 234**
Mean age, years	52.1	52.1	59.1
Histological findings			
Scirrhous carcinoma	32 (24.1)	51 (29.0)	86 (36.7)
Solid-tubular carcinoma	68 (51.1)	57 (32.4)	57 (24.4)
Papillotubular carcinoma	19 (14.2)	49 (27.8)	74 (31.6)
Other type of carcinoma	14 (10.5)	19 (10.8)	17 ( 7.3)
Pathological nodal status			
N0	83 (62.4)	99 (56.3)	151 (64.5)
N1	31 (23.3)	58 (32.9)	59 (25.2)
N2-3	18 (13.5)	14 ( 8.0)	16 ( 6.8)
Unknown	1 (0.8)	5 (2.8)	8 ( 3.4)
Pathological stage			
0	5 (3.8)	7 ( 4.0)	16 ( 6.8)
I	79 (59.4)	96 (54.5)	86 (36.8)
II	24 (18.0)	45 (25.6)	95 (40.6)
III	15 (11.3)	20 (11.4)	23 ( 9.8)
IV	10 ( 7.5)	8 ( 4.5)	14 ( 6.0)

**Table 2:** The change in the surgical procedures over the three periods

	**1980 to 1989, n = 133**	**1990 to 1999, n = 176**	**2000 to 2009, n = 234**
Extended radical mastectomy^1^	6 (4.5)	4 ( 2.3)	0
Halsted’s radical mastectomy	54 (40.6)	15 ( 8.5)	1 ( 0.4)
Modified radical mastectomy^2^	72 (54.1)	125 (71.0)	148 (63.2)
Simple mastectomy	1 (0.7)	10 ( 5.7)	1 ( 0.4)
Breast-conserving surgery	0	22 (12.5)	84 (35.9)

^1^Radical mastectomy with intrapleural *en bloc* resection of the internal mammary lymph node by sternal splitting.

^2^Mastectomy preserving the pectoral muscle.

**Figure 1:**
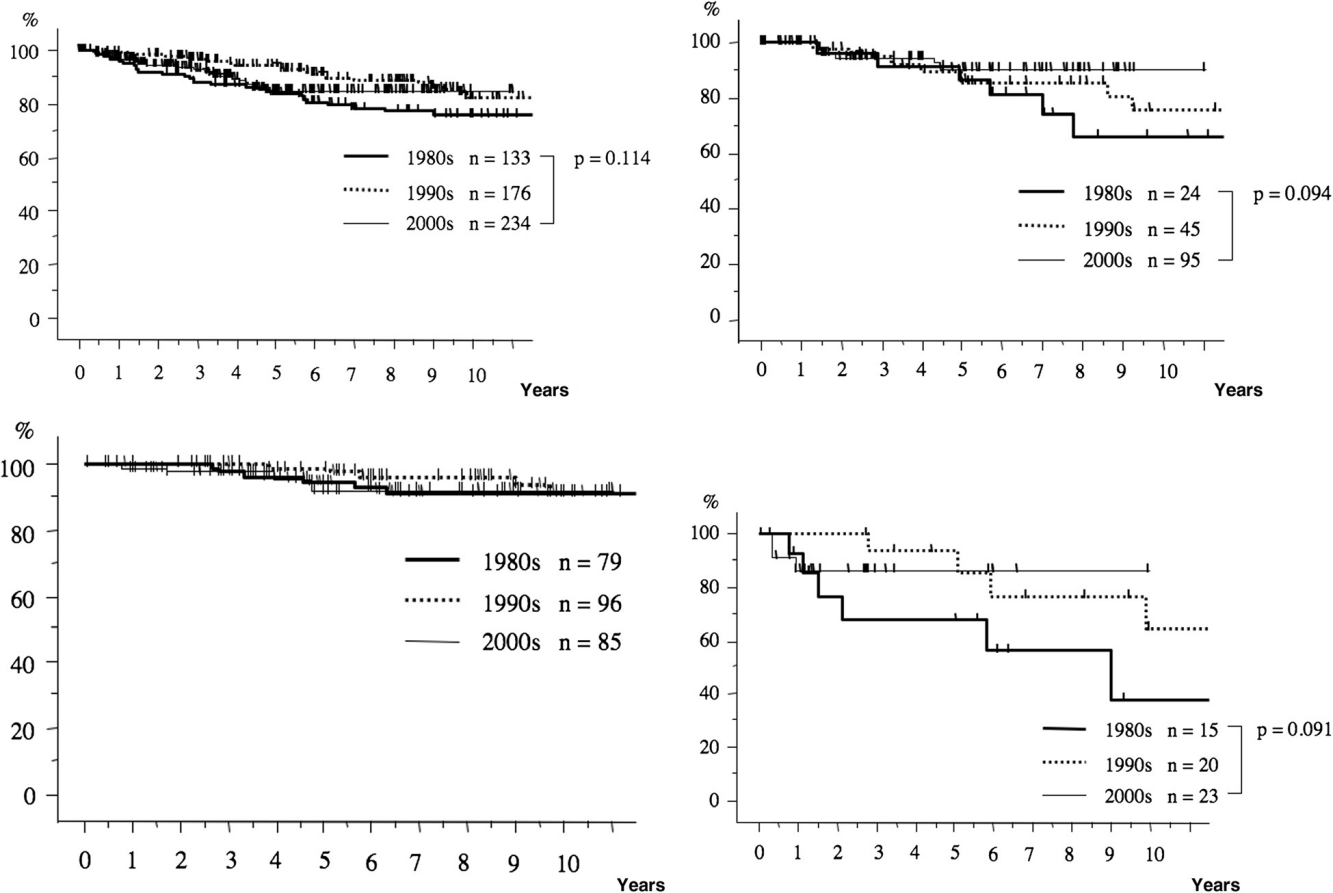
**The overall survival curves of the patients with breast cancer over the three periods.** The overall survival in each period was analyzed by the Kaplan-Meier method. The postoperative 10-year survival rate during the three periods was 75.9%, 83.5%, and 84.9%, respectively.

**Figure 2:**
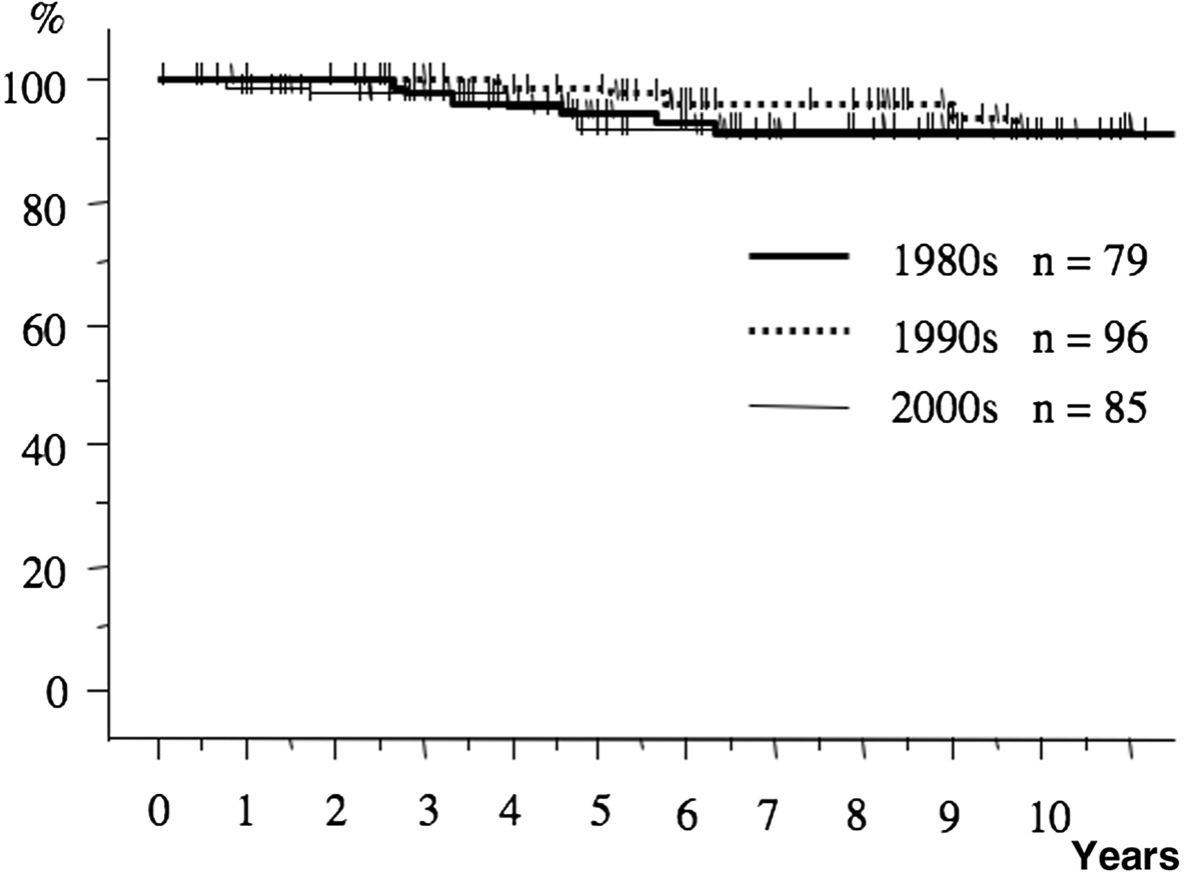
**The overall survival curves of the patients with stage I breast cancer over the three periods.** The 10-year survival rate in patients with pathological stage I breast cancer during each period was 91.2%, 91.1%, and 92.0%, respectively.

**Figure 3:**
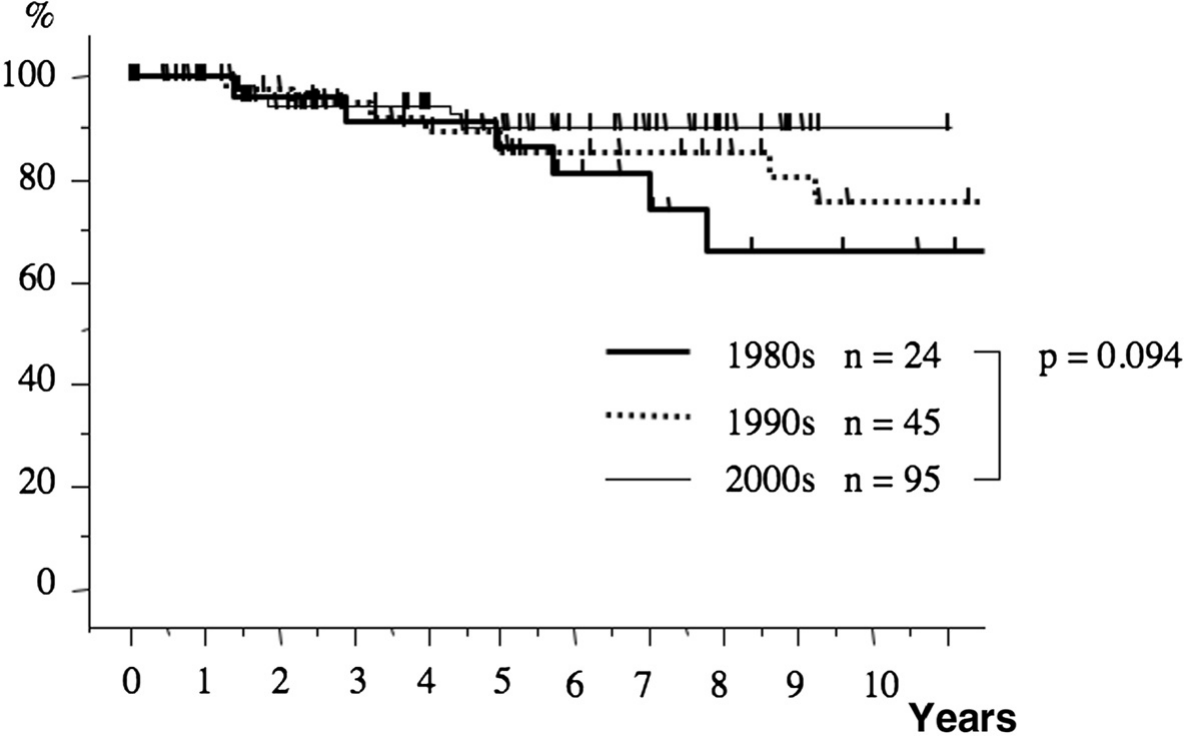
**The overall survival curves of the patients with stage II breast cancer over the three periods.** The 10-year survival rate of patients with stage II disease during the three periods tended to increase from 66.2% to 75.7%, and finally to 90.7% (*P* = 0.094).

**Figure 4:**
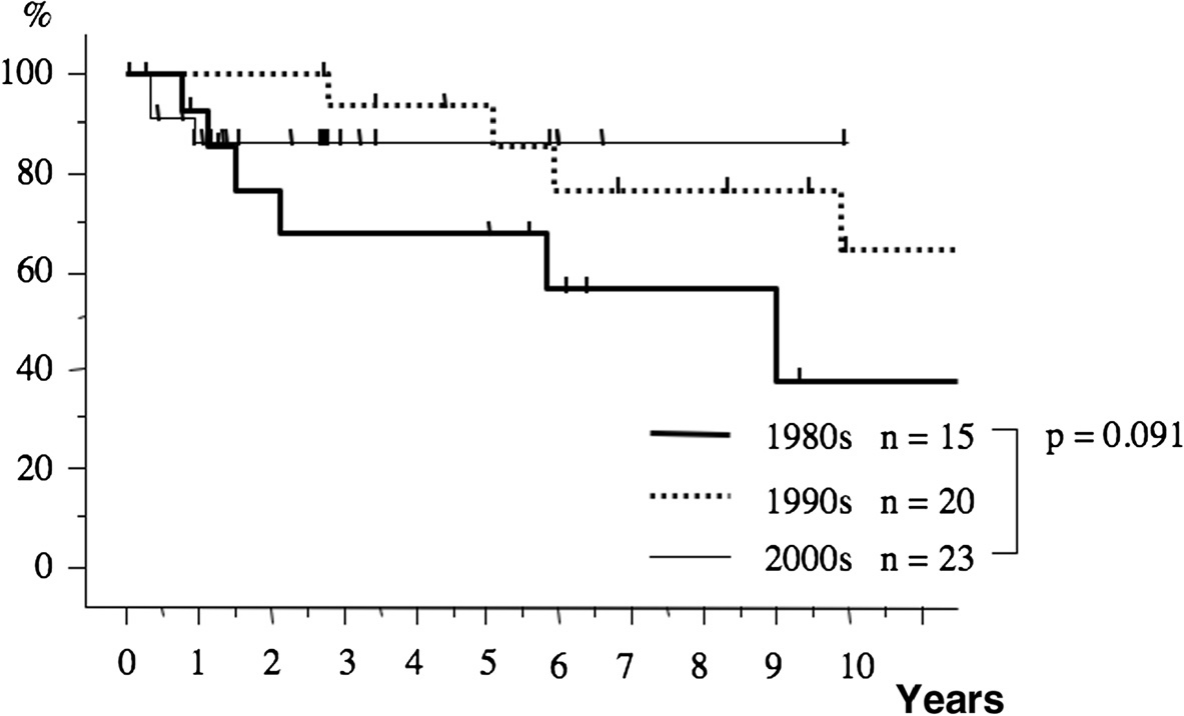
**The overall survival curves of the patients with stage III breast cancer in accordance with the period.** The prognosis of patients with stage III disease in the three periods found a tendency toward improvement, from 37.8%, to 64.2%, and finally to 86.5%, respectively (p = 0.091).

The postoperative 10-year survival rates during the three periods were 75.9%, 83.5%, and 84.9%, respectively (Figure 1). The 10-year survival rate in patients with pathological stage I breast cancer during each period was 91.1%, 91.2%, and 92.0%, respectively (Figure 2). The 10-year survival rates of patients with stage II disease during the three periods tended to increase from 66.2% during the first period to 75.7% and then to 90.7% (Figure 3). The prognosis of stage III disease in the three periods also showed a tendency toward improvement, rising from 37.8% to 64.2%, and finally to 84.5% (Figure 4).

## Discussion

The treatment strategy for breast cancer has drastically changed over the past three decades. In the 1980s, after the development of a surgical technique known as the radical mastectomy, surgeons considered breast cancer to be a curable disease. However, in recent years, the surgical procedures have become less invasive because there has been a paradigm shift in the perceptions of the disease and its treatment. In the National Surgical Adjuvant Breast and Bowel Project (NSABP)04 study, radical surgery with resection of the pectoral muscle (Halstead mastectomy) was compared with total mastectomy with and without radiotherapy [8]. With respect to the disease-free and overall survival rates, extended surgery was less advantageous than total mastectomy. In addition, in the NSABP06 study, patients with a tumor diameter of 40 mm or less were divided into three groups, in which patients were treated with a total mastectomy or partial resection of the breast with or without radiotherapy, and the results were compared [9]. There were no significant differences between the three groups in either disease-free or overall survival rates. Furthermore, given the patient radiotherapy after a partial mastectomy significantly inhibited recurrence in the residual breast. Veronesi *et al*. compared the Halsted mastectomy procedure with breast-conserving surgery plus radiotherapy in patients with invasive ductal carcinoma measuring 20 mm or less in diameter [10]. Although the local recurrence rate was significantly higher for the latter than for the former, there were no significant differences in the overall survival rates between the two procedures. Based on the results of these clinical trials, breast-conserving surgery has become the preferred surgical procedure. If the size of the tumor is large, a mastectomy will be necessary, unless pre-operative neoadjuvant chemotherapy can shrink the tumor sufficiently to allow for breast-conserving surgery. The proportion of breast-conserving therapy in our department reached 50% during the past 5 years.

Axillary lymph-node metastasis is one of the most important prognostic factors for the breast cancer [11]. Axillary dissection for the purpose of local control and axillary staging was previously a standard technique; however, with the changes in the importance of surgery as a local therapy, the importance of axillary dissection has also been changing, from local control to staging of the disease. The NSABP study investigated the usefulness and safety of sentinel lymph-node biopsy (SLNB), and found that when the SLNB did not reveal any metastasis axillary lymph-node dissection increased the incidence of complications without improving the prognosis [12]. Based on clinical evidence, SLNB has been commonly used for patients with breast cancer. In the future, the significance of axillary lymph-node dissection in patients with metastasis who have undergone SLNB should be clarified.

Currently, drug therapy plays a principal role in breast-cancer treatment, and the remarkable advances in drug therapy have led to a decrease in the breast-cancer-related mortality rate in Europe and the USA. Previously, drug therapy was selected in accordance with the ‘relapse risk classification’, which was based on the presence or absence of lymph-node metastasis. However, it has recently been recommended that the application of target therapy based on hormone sensitivity (ER status) and HER2 status should be considered for the initial management of patients. With regard to hormone therapy, the drug selection depends on the female hormonal environments (pre- and post-menopausal conditions). In pre-menopausal patients, ovarian functional suppression (luteinizing hormone (LH)-releasing hormone analogs) and ER blockers (tamoxifen) are [13]. In our hospital, the use of LH-releasing hormone analogs was started in 1995 In post-menopausal patients, the development of aromatase inhibitors led to a switch from a conventional standard agent, tamoxifen, to aromatase inhibitors in the early 2000s [14].

With regard to chemotherapy, anthracyclines or taxanes are recommended as first-line agents. As a significant benefit of anthracycline-based chemotherapy compared with CMF (cyclophosphamide, methotrexate, fluorouracil) has been clearly shown, anthracycline-based regimens such as FEC (fluorouracil, epirubicin, cyclophosphamide) or CAF (fluorouracil, doxorubicin, cyclophosphamide) are currently used as standard treatment [15]. In recent years, anthracycline-based chemotherapy was followed by taxanes when the risk of relapse was high. Furthermore, trastuzumab, which acts on HER2 receptors, has markedly improved the prognosis of patients with HER2-positive breast cancer, further changing drug therapy regimes for breast cancer [16]. We started to use trastuzumab for patients with HER2-positive breast cancer form the early 2000s.

## Conclusion

In summary, the treatment of breast cancer has changed greatly over the past three decades. In the present study, we noted that the survival of patients with stage II and III disease has improved. The prognosis of patients with recurrent breast cancer also improved over time after the introduction of aromatase inhibitors and trastuzumab, and the survival improvement was especially apparent in patients with ER-positive and/or HER2-positive tumors. Along with the recent advances in drug therapy, the surgical treatments have been updated and are now based on drug therapy-related modifications.

## Competing interests

The authors declare that they have no competing interests.

## Authors’ contribution

TH conceived of the study, and drafted the manuscript. YN participated in the study and performed the statistical analysis. SM, SS, MT, YS, HS, MN, HU, TS and FT participated in the study and coordination. All authors read and approved the final manuscript.
